# Effect of Acute Stressor and Serotonin Transporter Genotype on Amygdala First Wave Transcriptome in Mice

**DOI:** 10.1371/journal.pone.0058880

**Published:** 2013-03-11

**Authors:** Christa Hohoff, Ali Gorji, Sylvia Kaiser, Edith Willscher, Eberhard Korsching, Oliver Ambrée, Volker Arolt, Klaus-Peter Lesch, Norbert Sachser, Jürgen Deckert, Lars Lewejohann

**Affiliations:** 1 Department of Psychiatry, University of Muenster, Muenster, Germany; 2 Institute of Neurophysiology, University of Muenster, Muenster, Germany; 3 Department of Behavioural Biology, University of Muenster, Muenster, Germany; 4 Otto Creutzfeldt Center for Cognitive and Behavioral Neuroscience, University of Muenster, Muenster, Germany; 5 Integrated Functional Genomics (IFG), Core Unit of the Interdisciplinary Centre of Clinical Research (IZKF) Muenster, Muenster, Germany; 6 Institute of Bioinformatics, University of Muenster, Muenster, Germany; 7 Department of Psychiatry, University of Wuerzburg, Wuerzburg, Germany; Radboud University, The Netherlands

## Abstract

The most prominent brain region evaluating the significance of external stimuli immediately after their onset is the amygdala. Stimuli evaluated as being stressful actuate a number of physiological processes as an immediate stress response. Variation in the serotonin transporter gene has been associated with increased anxiety- and depression-like behavior, altered stress reactivity and adaptation, and pathophysiology of stress-related disorders. In this study the instant reactions to an acute stressor were measured in a serotonin transporter knockout mouse model. Mice lacking the serotonin transporter were verified to be more anxious than their wild-type conspecifics. Genome-wide gene expression changes in the amygdala were measured after the mice were subjected to control condition or to an acute stressor of one minute exposure to water. The dissection of amygdalae and stabilization of RNA was conducted within nine minutes after the onset of the stressor. This extremely short protocol allowed for analysis of first wave primary response genes, typically induced within five to ten minutes of stimulation, and was performed using Affymetrix GeneChip Mouse Gene 1.0 ST Arrays. RNA profiling revealed a largely new set of differentially expressed primary response genes between the conditions acute stress and control that differed distinctly between wild-type and knockout mice. Consequently, functional categorization and pathway analysis indicated genes related to neuroplasticity and adaptation in wild-types whereas knockouts were characterized by impaired plasticity and genes more related to chronic stress and pathophysiology. Our study therefore disclosed different coping styles dependent on serotonin transporter genotype even directly after the onset of stress and accentuates the role of the serotonergic system in processing stressors and threat in the amygdala. Moreover, several of the first wave primary response genes that we found might provide promising targets for future therapeutic interventions of stress-related disorders also in humans.

## Introduction

Acute stressors such as unexpected and potentially threatening changes of an individual’s environment usually provoke immediate stress responses to adapt, which are accompanied by and actuating a number of physiological processes [1]. However, the way an individual responds to stress may vary depending on a wide range of environmental as well as genetic factors. A promising candidate for a further elucidation of these processes is the serotonergic system which is strongly involved in stress response and adaptation. A key regulator of serotonergic activity in the central nervous system is the serotonin transporter (5-HTT) that has also been linked to inappropriate regulation of the stress response, for example in anxiety- and stress-related personality traits and neuropsychiatric disorders [2], [3].

The human gene encoding the 5-HTT (Slc6A4) is modulated by a length variation of a repetitive element in its upstream regulatory region commonly composed of either 14 (short allele) or 16 (long allele) repeated elements [4], [5], [6] The short variant of the polymorphism causes a reduction in the transcriptional efficiency of the 5-HTT promoter, resulting in decreased 5-HTT expression and therefore a reduced 5-HT reuptake from the synaptic cleft into the presynaptic cell compared to the long variant [6], [7]. The low-expressing 5-HTT short variant has been implicated in higher scores of neuroticism and is associated to a heightened trait anxiety/dysphoria and an exaggerated response to fear as well as environmental stress [2], [8], [9], [10]. The evidence of a connection between mood disorders and genetic variation of the 5-HTT led to the generation of 5-HTT knockout mice with a targeted inactivation of 5-HTT function [11]. This genetic modification led to the identification of fundamental phenotypic changes, ranging from increased anxiety- and depression-related behaviors, gene expression differences to altered dendritic morphology (reviewed by [12]). Further, a growing literature indicates altered stress reactivity and impaired abilities to cope with or adapt to stress in 5-HTT knockout mice [13], [14], involving also differences in gene expression [15], [16].

Within the central nervous system, 5-HT nerve terminals are particularly present in stress-related brain regions like amygdala, hippocampus, striatum and cortex [17]. Of these, the amygdala is one of the core systems driving emotional processes and vigilance by evaluating the significance of a stressor and triggering the most adequate emotional response based upon previous experiences as well as upon genetic predispositions [18]. A strong link exists between altered amygdala function and psychopathology, e.g. association of the short 5-HTT variant with a relative exaggeration in the response of the amygdala to anxiety-provoking or stress-related stimuli [3], [18], [19], [20], [21], [22]. Further, changes in gene expression accompanying stress reactivity and coping also affect the amygdala, both after acute stress [23], [24], [25], [26] as well as after chronic stress [27], [28]. However, nothing is known about rapid changes in amygdala gene expression following immediately after an acute stressor in case of individuals with an imbalanced serotonergic system that are prone to inappropriate stress response. We hypothesized basal differences in anxiety-related behavior of mice as previously described between 5-HTT genotypes [13], [29] and differences in mRNA profiles that are dependent on the condition (acutely stressed vs. control) and the genotype (5-HTT knockout vs. wild-type). Such differences between the genotypes in their first wave response might prove to be extremely valuable for explaining genotype-dependent differences in coping with stress.

The present study thus aimed to explore immediate changes in genome-wide amygdala mRNA profiles in response to an acute stressor using the 5-HTT knockout mouse model. We observed basal differences in anxiety-related behavior as well as differing transcriptomes between acutely stressed compared to control mice already a few minutes after the stressor together with differences depending on 5-HTT genotype reflecting 5-HTT level-based changes in emotion processing and stress coping.

## Methods

### Animals and Housing Conditions

5-HTT knockout mice were generated on a C57BL/6J background [11]. Initially a total of 16 knockout (−/−, *KO*) and 14 wild-type (+/+, *WT*) male mice, descendants from heterozygous parents from the internal stock bred at the University of Muenster, were included for behavioral analysis. Sample sizes were reduced for hormonal analysis (see below) and further cut down for microarray analysis. Genotypes were identified by gel electrophoresis of DNA-fragments of either 225 bp (*WT*) or 272 bp (*KO*) [11]. Testing animals were weaned at 21 days of age and maintained in sibling groups of 3 to 5 male mice until they were transferred to single housing at an age of 5 months, 2 months prior to behavioral testing. The housing room was maintained at a 12 h light/dark cycle (lights on at 8.00 h) at a temperature of 22±3°C.

The presented work complies with current regulations covering animal experimentation in Germany and experiments were approved by the competent local authority, North Rhine-Westphalia State Agency for Nature, Environment and Consumer Protection (LANUV NRW, Permit number: 8.87–50.10.46.08.090), and all efforts were made to minimize suffering.

### Elevated Plus-maze Test (EPM)

At the age of 7 months ±14 days the mice were tested in the EPM. The plus shaped apparatus was elevated 50 cm above the ground and illuminated by an overhead bulb (200 lux in the center of the EPM). The maze comprised four arms (5 cm×30 cm) extending from a central platform (5 cm×5 cm), two opposing arms were enclosed with 20 cm high walls and the other two opposing arms were unshielded. The mice were placed for 1 min in an empty cage prior to testing to assure comparable amounts of arousal. Each mouse was placed on the central platform facing a shielded arm and was then allowed to freely explore the apparatus for 10 min. The movements of the animals were recorded by a digital tracking system (www.phenotyping.com/digital.html) assessing path-length, arm entries and time spent within the arms. Statistics were calculated using the R software package v2.15.0 (www.r-project.org). Deviation from normal distribution was analyzed by one-sample Kolmogorov–Smirnov test and in addition Levene’s test for homogeneity of variance was calculated. As a consequence behavioral data were analyzed using the non-parametric unpaired Wilcoxon exact rank sum test (two-sided) since the data sets showed non-Gaussian distributions that could not reasonably be transformed to normal distribution.

### Acute Stress and Tissue Sampling

Two weeks after testing in the EPM, mice were subjected to two different conditions, either acute stress or control condition. Groups sizes were *KO*: n_acute stress_ = n_control_ = 8, *WT*: n_acute stress_ = 8, n_control_ = 6. Sampling was conducted in a standardized way within 9 min involving four experimenters who were coordinated with the help of an in-house developed software that prompted every single step of the time-critical procedure ("Talkative Timer", www.phenotyping.com/talktimer.html, available on demand). The protocol started with an experimenter entering the housing room and transferring the cage of the experimental animal to an adjacent room where half of the mice were left untouched in their cages for 1 min (control) and the other half was placed into a bucket filled with water (∼21°C) for 1 min (acute stress). Afterwards all mice were anaesthetized by isoflurane for 40 sec, weighed, and decapitated exactly 160 sec from the start of the protocol. Trunk-blood was collected during the following 40 sec using heparinized capillaries, centrifuged (5 min at 14800 × g) and plasma was stored at −20°C for later evaluation. The brain of each mouse was dissected from the skull within 105 sec, starting 175 sec after the first step of the procedure. It was placed into ice cold (4°C) artificial cerebrospinal fluid (124 mM NaCl, 4 mM KCl, 1 mM CaCl2, 1.24 mM NaH2PO4, 1.3 mM MgSO4, 26 mM NaHCO3, 10 mM glucose; pH 7.4) for 60 sec prior to dissection of both amygdalae within 90 sec.

Homogenization of the amygdalae started after 430 sec from the protocol start by comminuting tissue in 1.5 ml eppendorf tubes using low volume filter tips (Type J, Sarstedt). After 60 sec 100 µl 2X Nucleic Acid Purification Lysis Solution (Applied Biosystems by Life Technologies) was added to further homogenize and effectively lyse tissue for 50 sec. Within this time cellular reactions were stopped, particularly all RNases were immediately inactivated and all RNAs precipitated to prevent degradation. Altogether 540 sec after the start of the protocol the procedure was finished and the samples were stored at −20°C for later RNA extraction.

### Corticosterone Assay

Plasma corticosterone concentrations were determined by enzyme linked immunosorbent assay (EIA, DE4164, Demeditec Diagnostics GmbH, Kiel, Germany) according to the manufacturer’s recommendations. All standards, samples, and controls were run in duplicate concurrently. The intra- and inter-assay coefficients of variation were 3.3% and 6.0%, respectively. Corticosterone titers were analyzed using the R software package (see above) by an ANOVA in a two by two factorial design with genotype and treatment as between subject factors.

### Microarray Analysis and Bioinformatics

Total RNA was extracted from the amygdala samples using the ABI 6100 Nucleic Acid PrepStation with Total RNA Chemistry according to the manufacturer’s instructions (Applied Biosystems) including preceding Proteinase K digestion (0.4 mg/sample at RT). RNA concentrations were calculated by measurement of 260/280 nm absorbance (Eppendorf BioPhotometer; RNA program): Samples below 30 ng/µl were concentrated by Microcon centrifugal filter devices (YM-3) as recommended by the manufacturer (Millipore). RNA quantity and quality were additionally determined using Agilent Bioanalyzer 2100 with Eukaryote Total RNA Nano Chip Assays and 2100 Expert software version B.02.06 (all Agilent Technologies). RNA quantity and quality threshold was set to 30 ng/µl and RNA Integrity Number (RIN) = 8. Altogether 20 samples were chosen (5 biological replicates per genotype and condition) fitting these recommendations with averaged concentration of 60 ng/µl (range 30–84) and RIN of 9.13 (range 8.2–9.7). Three µl per RNA sample were used for subsequent gene expression analysis of 28853 genes using GeneChip Mouse Gene 1.0 ST Arrays (Affymetrix, MoGene-1_0-st). The 20 microarray datasets (CEL files) were analyzed with ArrayAssist Software (Stratagene/Agilent). ExonRMA algorithm was used for summarization and normalization. After that data was variance stabilized, transformed on logarithmic scale and quality control was performed. All microarray data in this publication are MIAME compliant, have been deposited in NCBI's Gene Expression Omnibus [30] and are accessible through GEO Series accession number GSE40393 (http://www.ncbi.nlm.nih.gov/geo/query/acc.cgi?acc=GSE40393). Differentially expressed genes were ascertained by unpaired t-tests in the four genotype by condition groups resulting in four different comparisons. Within the genotypes the conditions acute stress (*stress*) versus control (*con*) were compared: *KO stress* vs. *con* and *WT stress* vs. *con*. Further, genotype comparisons were performed within the control condition (*con KO* vs. *WT*) as well as within the acute stress condition (*stress KO* vs. *WT*). A correction for false discovery rate was not applied because this proved overly stringent given the high number of genes that were included and therefore was likely to produce false negative results. Instead, we applied a combined criterion for true positive expression differences of a fold difference >1.5 and a P-value <0.05.

Gene functional annotation and enrichment analyses were performed using the web-based tool DAVID Bioinformatics Resources 6.7 (Database for Annotation, Visualization and Integrated Discovery v6.7; http://david.abcc.ncifcrf.gov/home.jsp [31], [32]). More specifically, own gene lists of group comparisons *KO stress* vs. *con*, *WT stress* vs. *con*, *con KO* vs. *WT*, and *stress KO* vs. *WT* with gene fold differences >1.5 and P-values <0.05 were uploaded and submitted to DAVID together with identifier Affymetrix_exon_gene_ID and pre-built Affymetrix Exon Background (MoGene-1_0-st-v1). For identification of groups with similar biological meaning that are enriched in own gene lists compared to the background, data were analyzed with Functional Annotation Clustering using default setting (medium) for classification stringency. Results were narrowed down on significant annotation clusters with Group Enrichment Scores ≥1.3, EASE scores (P-values) for individual term members ≤0.05, and fold enrichment ≥1.5, as well as low family-wise false discovery rates (Benjamini multiple testing correction P-values ≤0.15). In addition, exploratory pathway analyses were performed with own gene lists of group comparisons using the Kyoto Encyclopedia of Genes and Genomes (KEGG) database implemented in DAVID.

The validity of the microarray results was subsequently tested via commercial quantitative real-time Taqman PCR assays for several candidate genes: one or two validated genes (no pseudo or predicted genes) with fold changes >1.75 per group comparison representing different annotation clusters and pathways, respectively, and Gapdh as endogenous control. PCRs were set according to the manufacturer’s instructions (Applied Biosystems) on a Tecan Freedom EVO 150 and relative quantification was analyzed using the 7900 HT system with software SDS 2.1.1 (Applied Biosystems).

## Results

### Elevated Plus-maze (EPM)

**Figure 1:**
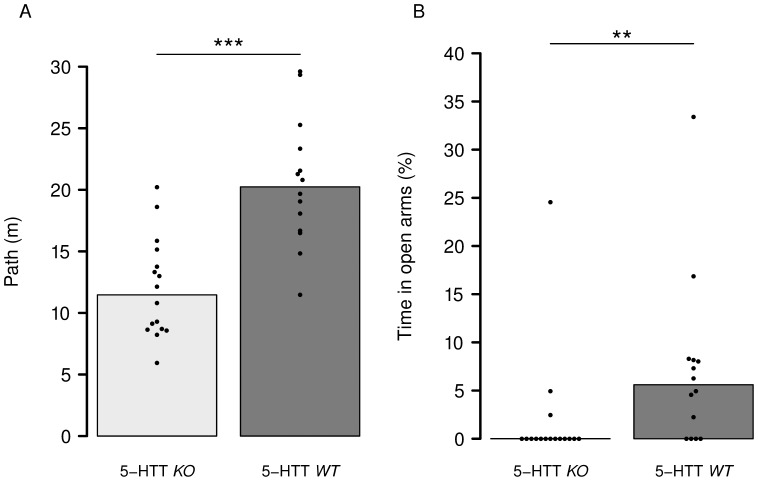
Elevated plus-maze test. a) Activity-related measures exemplified as the path length covered during the test. Bars represent the median values and the dots represent the data of each individual animal. b) Anxiety-related behavior measured as the proportion of time spent in open arms. Serotonintransporter knockout mice (5-HTT *KO*, n = 17) were less active and spent a lower proportion of time on open arms indicating increased anxiety compared to wild-type (5-HTT *WT*, n = 14) mice. Statistics: Wilcoxon exact rank sum test: ** = P<0.01, *** = P<0.001.

General activity in the EPM measured as total number of arm entries and path length differed between *WT* and *KO* mice with the latter being less active (Wilcoxon Test, path length: W = 20, P<0.0001, Fig. 1a; total arm entries: W = 31, P = 0.0008). Consequently, anxiety-related measures of number of open arm entries and time spent in open arms of the EPM were calculated as percent. *WT* mice entered a significantly higher percentage of open arms (Wilcoxon Test, W = 53.5, P = 0.0077) and spent significantly more percentage of time in the open arms as *KO* mice (Wilcoxon Test, W = 50, P = 0.0047, Fig. 1b).

### Corticosterone

**Figure 2:**
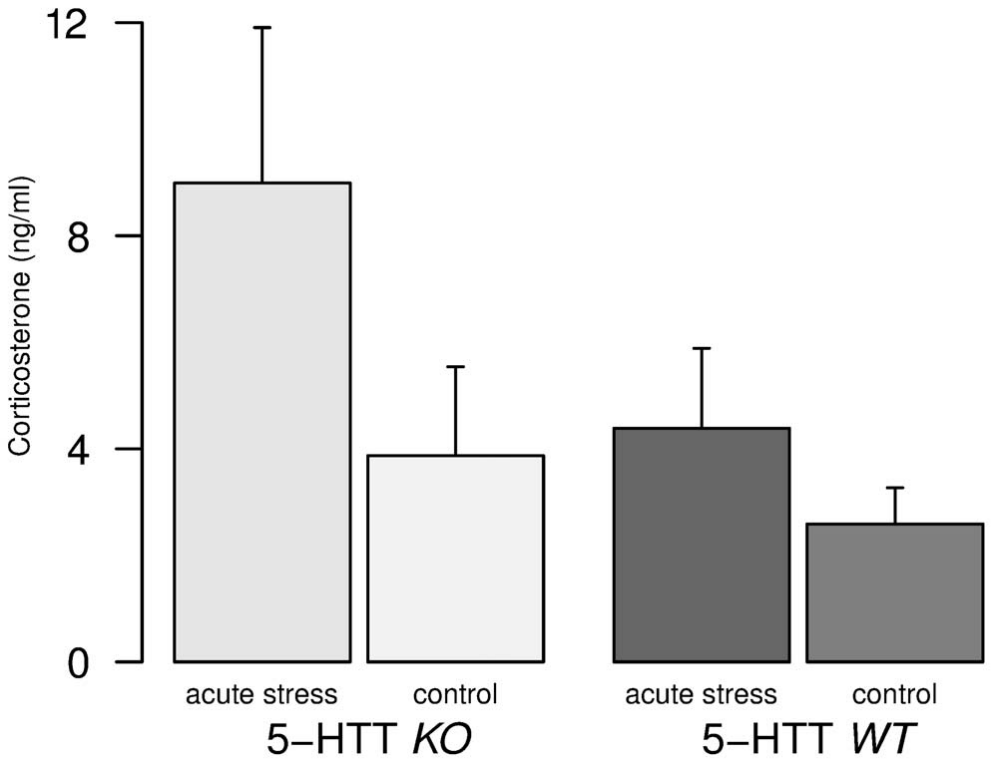
Corticosterone stress hormone concentration. Trunk blood samples were taken 130 sec after the mice were either being placed for 1 min in 21°C water (acute stress) or left untouched in the home-cage for 1 min (control). Bars represent the mean concentration, whiskers express SEM. An ANOVA revealed no significant effect of genotype (F_1,26_ = 2.36, P = 0.14) but a trend for increased stress hormone concentrations in mice subjected to acute stress (F_1,26_ = 3.21, P = 0.085).

Plasma corticosterone titers (Fig. 2) were at basal levels in the control condition in both genotype groups (mean *KO*: 3.87±1.67 (SEM) ng/ml, mean *WT*: 2.59±0.68 (SEM) ng/ml). Acute stress overall led to an increase of stress hormone concentration in both genotypes (mean *KO*: 8.99±2.92 (SEM) ng/ml, mean *WT*: 4.39±1.5 (SEM) ng/ml).

ANOVA revealed a trend for increased stress hormone concentrations in mice subjected to acute stress (F_1,26_ = 3.21, P = 0.085) but no effect of genotype (F_1,26_ = 2.36, P = 0.14) and there was no significant interaction of genotype by stress (F_1,26_ = 0.68, P = 0.42).

### Microarray Analysis

Microarray analysis disclosed 80 differentially expressed genes between the conditions acute stress and control. Most of them, that is 77 genes, were upregulated immediately after the stressor, only 3 genes were downregulated. Closer inspection of the impact of acute stress vs. control in the different 5-HTT genotype groups revealed no overlap of genes in the expression profiles. About half of the genes were differentially expressed in *WT* mice after the acute stressor (38 genes: 36 up-, 2 downregulated) and the other half in *KO* mice (42 genes: 41 up-, 1 downregulated). Direct comparison of both genotypes within the acute stress condition indicated 90 genes differentially expressed, 36 of them upregulated in *WT* mice and the other 54 upregulated in *KO* mice. In contrast, within the control condition only slight differences appeared between both genotypes with only 15 differentially expressed genes, 9 of them upregulated in *WT* and 6 in *KO* mice (for detailed gene lists concerning all group comparisons see Table S1).

### Functional Annotation Clustering and KEGG Pathway Analysis by DAVID

In the next step the resulting microarray group gene lists were functionally categorized using DAVID. Significant annotation clusters were detected in the groups *acute stress* vs. *control* including the terms neurogenesis and cation binding as well as terms like glycoprotein, membrane, and transport (for a complete list of clusters and enriched terms in all gene groups see Table 1). Again closer inspection of the impact of acute stress within the different 5-HTT genotype groups revealed no overlap of clusters: the first terms (neurogenesis, cation binding) were observed only in *WT* mice and the latter terms (glycoprotein, membrane, transport) only in *KO* mice. The direct genotype comparison *WT* vs. *KO* within the stress condition revealed three clusters with the terms calcium ion binding, cilium/cell projection, and cell projection part. Of these, terms including ion binding (metal ion/cation/calcium binding) were enriched in upregulated genes of *WT* mice, while the other terms including cilium/axoneme/cilium part/cell projection part were enriched in upregulated genes of *KO* mice (Table 1). In contrast, no functional clusters reaching our criteria of significance were detected in the genotype comparison *WT* vs. *KO* within the control condition.

**Table 1:** Significant functional annotation clusters in different gene groups related to condition (*acute stress* or *control*) and 5-HTT genotype (*WT* or *KO*) acquired by DAVID tool.

Cluster	Scores	Category	Enrichment Term	# (%) Genes	P-Value
Group: *WT stress* vs. *con* (Total: 38 genes)					
1	1.76	SP_PIR_KEYWORDS	neurogenesis	4 (10.5)	0.003
2	1.29	GOTERM_MF_FAT	GO:0043169∼cation binding	17 (44.7)	0.004
		GOTERM_MF_FAT	GO:0043167∼ion binding	17 (44.7)	0.004
Group: *KO stress* vs. *con* (Total: 42 genes)					
1	6.03	SP_PIR_KEYWORDS	glycoprotein	26 (61.9)	1.53E−09
		SP_PIR_KEYWORDS	Secreted	16 (38.1)	8.70E−08
		UP_SEQ_FEATURE	glycosylation site:N-linked (GlcNAc…)	24 (57.1)	2.18E−07
		SP_PIR_KEYWORDS	signal	21 (50.0)	3.73E−07
		GOTERM_CC_FAT	GO:0044421∼extracellular region part	13 (31.0)	7.92E−07
		UP_SEQ_FEATUR	signal peptide	21 (50.0)	1.91E−06
		GOTERM_CC_FAT	GO:0005576∼extracellular region	17 (40.5)	5.43E−06
		GOTERM_CC_FAT	GO:0005615∼extracellular space	10 (23.8)	8.24E−06
		SP_PIR_KEYWORDS	disulfide bond	17 (40.5)	1.81E−05
		UP_SEQ_FEATURE	disulfide bond	17 (40.5)	4.06E−05
2	2.32	GOTERM_CC_FAT	GO:0031226∼intrinsic to plasma membrane	8 (19.0)	8.56E−04
		GOTERM_CC_FAT	GO:0005887∼integral to plasma membrane	7 (16.7)	0.004
		GOTERM_CC_FAT	GO:0005886∼plasma membrane	16 (38.1)	0.011
3	2.27	UP_SEQ_FEATURE	topological domain:Extracellular	15 (35.7)	2.80E−04
		UP_SEQ_FEATURE	topological domain:Cytoplasmic	16 (38.1)	0.001
		SP_PIR_KEYWORDS	membrane	22 (52.4)	0.002
		UP_SEQ_FEATURE	transmembrane region	19 (45.2)	0.003
		SP_PIR_KEYWORDS	transmembrane	20 (47.6)	0.006
		GOTERM_CC_FAT	GO:0005886∼plasma membrane	16 (38.1)	0.011
4	1.75	GOTERM_MF_FAT	GO:0022803∼passive transmembrane transporter activity	6 (14.3)	0.002
		GOTERM_MF_FAT	GO:0015267∼channel activity	6 (14.3)	0.002
		SP_PIR_KEYWORDS	voltage-gated channel	4 (9.5)	0.003
		SP_PIR_KEYWORDS	ionic channel	5 (11.9)	0.004
		SP_PIR_KEYWORDS	transport	10 (23.8)	0.005
Group: *con KO* vs. *WT* (Total: 15 genes)					
–	–	–	–	–	–
Group: *stress KO* vs. *WT* (Total: 90 genes)					
1	2.26	GOTERM_MF_FAT	GO:0005509∼calcium ion binding	11 (12.1)	7.27E−04
		SP_PIR_KEYWORDS	calcium	10 (11.0)	8.87E−04
2	2.21	GOTERM_CC_FAT	GO:0005929∼cilium	5 (5.5)	6.62E−04
		GOTERM_CC_FAT	GO:0044463∼cell projection part	5 (5.5)	0.002
		SP_PIR_KEYWORDS	cilium	4 (4.4)	0.002
		GOTERM_CC_FAT	GO:0044441∼cilium part	3 (3.3)	0.006
		GOTERM_CC_FAT	GO:0042995∼cell projection	7 (7.7)	0.007
3	1.35	GOTERM_CC_FAT	GO:0044463∼cell projection part	5 (5.5)	0.002
Group: *stress KO* vs. *WT*, upregulated in *KO* (Total: 54 genes)					
1	1.82	GOTERM_CC_FAT	GO:0005929∼cilium	5 (9.3)	1.64E−04
		SP_PIR_KEYWORDS	cilium	4 (7.4)	7.47E−04
		GOTERM_CC_FAT	GO:0005930∼axoneme	3 (5.6)	0.003
		GOTERM_CC_FAT	GO:0044441∼cilium part	3 (5.6)	0.003
		GOTERM_CC_FAT	GO:0044463∼cell projection part	4 (7.4)	0.005
Group: *stress KO* vs. *WT*, upregulated in *WT* (Total: 36 genes)					
1	2.55	GOTERM_MF_FAT	GO:0046872∼metal ion binding	11 (29.7)	5.44E−04
		GOTERM_MF_FAT	GO:0043169∼cation binding	11 (29.7)	5.90E−04
		GOTERM_MF_FAT	GO:0043167∼ion binding	11 (29.7)	6.59E−04
		SP_PIR_KEYWORDS	calcium	5 (13.5)	0.002
		GOTERM_MF_FAT	GO:0005509∼calcium ion binding	5 (13.5)	0.008

The different annotation clusters were represented by varying fractions of genes ranging from only about 5–10% up to 40–60% (overview: Table 1; details: Tables 2, 3, 4). The neurogenesis cluster, that comprised about 10% of all genes upregulated in *WT stress* vs. *con*, included genes like roundabout homolog 2 (Robo2), slit homolog 3 (Slit3), and sema domain 3E (Sema3e). All these genes were also included in the functionally highly overlapping KEGG pathway axon guidance (Table 2; details: Table 5), supporting the relevance of this cluster. The second cluster detected in *WTs* after stress, cation binding, had marginal enrichment score but represented about 45% of this group. It included genes like cadherin 9 (Cdh9), Slit3, calcium binding protein 1 (Cabp1), parvalbumin (Pvalb) and several receptors like glutamate receptor, ionotropic, NMDA2A (Grin2a), nuclear receptors of subfamiliy 4, group A, members 2 and 3 (Nr4a2, Nr4a3), and ryanodine receptor 1 (Ryr1). Again similar results were obtained from KEGG pathway analysis that revealed the calcium signaling pathway containing genes like Grin2a and Ryr1, however with EASE score reaching only trend significance (Table 2, 5).

**Table 2:** Genes included in functional annotation clusters in gene group of 5-HTT *WT* mice in conditions *acute stress* vs. *control* as acquired by DAVID tool.

Cluster	Transcript	Gene Title	Symbol	FC^1^	Effect	P-Value
neurogenesis	10440344	roundabout homolog 2 (Drosophila)	Robo2	1.79	up	0.014
neurogenesis	10519747	sema domain, immunoglobulin domain (Ig), short basic domain, secreted	Sema3e	1.77	up	0.025
neurogenesis	10481349	netrin G2///RIKEN cDNA 6530402F18 gene	Ntng2	1.7	up	0.003
neurogenesis, cation binding	10375175	slit homolog 3 (Drosophila)	Slit3	1.57	up	0.012
cation binding	10586591	carbonic anyhydrase 12	Car12	2.88	up	0.032
cation binding	10523483	PR domain containing 8	Prdm8	2.25	up	0.047
cation binding	10485745	anoctamin 3	Ano3	2.09	up	0.006
cation binding	10423230	cadherin 9	Cdh9	2.07	up	0.004
cation binding	10504838	nuclear receptor subfamily 4, group A, member 3	Nr4a3	2.06	up	0.020
cation binding	10556381	microtubule associated monoxygenase, calponin and LIM domain containing 2	Mical2	1.81	up	0.033
cation binding	10437629	glutamate receptor, ionotropic, NMDA2A (epsilon 1)	Grin2a	1.8	up	0.038
cation binding	10482772	nuclear receptor subfamily 4, group A, member 2	Nr4a2	1.73	up	0.039
cation binding	10548940	LIM domain only 3	Lmo3	1.7	up	0.002
cation binding	10532956	calcium binding protein 1	Cabp1	1.59	up	0.023
cation binding	10561561	ryanodine receptor 1, skeletal muscle	Ryr1	1.58	up	0.019
cation binding	10526038	matrix metallopeptidase 17	Mmp17	1.55	up	0.032
cation binding	10554819	malic enzyme 3, NADP(+)-dependent, mitochondrial	Me3	1.51	up	0.029
cation binding	10364792	polo-like kinase 5 (Drosophila)	Plk5	1.51	up	0.023
cation binding	10416800	LIM domain only 7	Lmo7	1.51	up	0.005
cation binding	10533751	phosphatidylinositol transfer protein, membrane-associated 2	Pitpnm2	1.51	up	0.027

1 Abbreviation: FC, fold change.

**Table 3:** Genes included in functional annotation clusters in gene group of 5-HTT *KO* mice in conditions *acute stress* vs. *control* as acquired by DAVID tool.

Cluster	Transcript	Gene Title	Symbol	FC^1^	Effect	P-Value
glycoprotein; transport	10454192	transthyretin	Ttr	5.92	up	0.024
membrane; transport	10356403	potassium inwardly-rectifying channel, subfamily J, member 13	Kcnj13	5.82	up	0.007
glycoprotein; plasma membrane; membrane	10527870	klotho	Kl	3.45	up	0.015
glycoprotein	10351224	coagulation factor V; similar to Murine coagulation factor V	F5	2.83	up	0.016
glycoprotein	10395389	sclerostin domain containing 1	Sostdc1	2.71	up	0.028
glycoprotein; transport	10478048	lipopolysaccharide binding protein	Lbp	2.38	up	0.040
membrane	10547191	transmembrane protein 72	Tmem72	2.1	up	0.033
glycoprotein	10347277	insulin-like growth factor binding protein 2	Igfbp2	2.06	up	0.032
glycoprotein	10569344	insulin-like growth factor 2	Igf2	2.05	up	0.043
glycoprotein; plasma membrane; membrane; transport	10436947	potassium voltage-gated channel, Isk-related subfamily, gene 2	Kcne2	2.04	up	0.035
glycoprotein; plasma membrane; membrane; transport	10495712	ATP-binding cassette, sub-family A (ABC1),member 4	Abca4	2.04	up	0.029
glycoprotein	10440091	collagen, type VIII, alpha 1	Col8a1	2.01	up	0.021
plasma membrane; membrane; transport	10436958	chloride intracellular channel 6	Clic6	1.98	up	0.003
glycoprotein; plasma membrane; membrane	10600024	G-protein-coupled receptor 50	Gpr50	1.95	up	0.050
plasma membrane; membrane	10602033	claudin 2	Cldn2	1.94	up	0.023
glycoprotein; plasma membrane; membrane	10428619	ectonucleotide pyrophosphatase/phosphodiesterase 2	Enpp2	1.92	up	0.031
glycoprotein; membrane; transport	10362104	solute carrier family 2 (facilitated glucosetransporter), member 12	Slc2a12	1.91	up	0.016
glycoprotein; plasma membrane; membrane	10456005	CD74 antigen	Cd74	1.87	up	0.021
glycoprotein; plasma membrane; membrane	10506301	leptin receptor	Lepr	1.86	up	0.034
glycoprotein; plasma membrane; membrane	10450154	histocompatibility 2, class II antigen A, alpha	H2-Aa	1.85	up	0.019
glycoprotein; plasma membrane; membrane	10466606	annexin A1	Anxa1	1.85	up	0.023
glycoprotein; plasma membrane; membrane	10381962	angiotensin I _raline_ne enzyme(peptidyl-dipeptidase A) 1	Ace	1.81	up	0.016
glycoprotein; membrane	10423049	prolactin receptor	Prlr	1.76	up	0.035
glycoprotein	10357870	_raline arginine-rich end leucine-rich repeat	Prelp	1.69	up	0.048
glycoprotein; plasma membrane; membrane	10362896	CD24a antigen	Cd24a	1.68	up	0.044
glycoprotein	10542993	paraoxonase 3	Pon3	1.66	up	0.036
plasma membrane; membrane; transport	10532839	transient receptor potential cation channel,subfamily V, member 4	Trpv4	1.65	up	0.039
glycoprotein; plasma membrane; membrane; transport	10538459	aquaporin 1	Aqp1	1.61	up	0.040
membrane	10422728	disabled homolog 2 (Drosophila)	Dab2	1.59	up	0.026
membrane	10373588	retinol dehydrogenase 5	Rdh5	1.58	up	0.015
glycoprotein	10344897	sulfatase 1	Sulf1	1.57	up	0.034
glycoprotein	10541354	alpha-2-macroglobulin	A2m	1.52	up	0.048
glycoprotein; membrane	10511180	matrix-remodelling associated 8	Mxra8	1.52	up	0.006
glycoprotein; membrane	10584653	C1q and tumor necrosis factor related protein 5	C1qtnf5	1.51	up	0.028
glycoprotein	10536220	collagen, type I, alpha 2	Col1a2	1.5	up	0.034
glycoprotein; plasma membrane; membrane; transport	10366391	potassium voltage gated channel, Shaw-related subfamily, member 2	Kcnc2	1.54	down	0.030

1 Abbreviation: FC, fold change.

**Table 4:** Genes included in functional annotation clusters in gene group of acutely stressed 5-HTT *KO* vs. *WT* mice as acquired by DAVID tool.

Cluster	Transcript	Gene Title	Symbol	FC^1^	P-Value
cilium; cell projection part	10567452	dynein, axonemal, heavy chain 3	Dnahc3	2.84	0.009
cilium; cell projection part	10362472	radial spoke head 4 homolog A (Chlamydomonas)	Rsph4a	1.99	0.010
calcium ion binding; cilium	10466606	annexin A1	Anxa1	1.85	0.030
calcium ion binding	10374777	epidermal growth factor-containing fibulin-like extracellular matrix protein 1	Efemp1	1.74	0.036
calcium ion binding	10399943	RIKEN cDNA 1110049B09 gene; Cadherin-like protein 28	CDHR3	1.74	0.037
cilium; cell projection part	10362896	CD24a antigen	Cd24a	1.70	0.008
calcium ion binding	10402211	fibulin 5	Fbln5	1.67	0.007
cilium; cell projection part	10494684	sperm associated antigen 17	Spag17	1.65	0.003
calcium ion binding	10457640	S100 calcium binding protein A11 (calgizzarin)	S100a11	1.61	0.047
calcium ion binding	10547227	ret proto-oncogene	Ret	1.59	0.006
calcium ion binding; cilium; cell projection part	10437629	glutamate receptor, ionotropic, NMDA2A (epsilon 1)	Grin2a	1.85	0.038
calcium ion binding	10423230	cadherin 9	Cdh9	1.84	0.023
calcium ion binding	10485745	anoctamin 3	Ano3	1.73	0.001
calcium ion binding	10532956	calcium binding protein 1	Cabp1	1.58	0.009
calcium ion binding; cilium	10430297	parvalbumin	Pvalb	1.58	0.036
upregulated in *KO*:					
cilium	10567452	dynein, axonemal, heavy chain 3	Dnahc3	2.84	0.009
cilium	10362472	radial spoke head 4 homolog A (Chlamydomonas)	Rsph4a	1.99	0.010
cilium	10466606	annexin A1	Anxa1	1.85	0.030
cilium	10362896	CD24a antigen	Cd24a	1.70	0.008
cilium	10494684	sperm associated antigen 17	Spag17	1.65	0.003
upregulated in *WT*:					
cation binding	10437629	glutamate receptor, ionotropic, NMDA2A (epsilon 1)	Grin2a	1.85	0.038
cation binding	10423230	cadherin 9	Cdh9	1.84	0.023
cation binding	10386705	ring finger protein 112	Zfp179	1.75	0.022
cation binding	10485745	anoctamin 3	Ano3	1.73	0.001
cation binding	10556381	microtubule associated monoxygenase, calponinand LIM domain containing 2	Mical2	1.69	0.047
cation binding	10532956	calcium binding protein 1	Cabp1	1.58	0.009
cation binding	10430297	parvalbumin	Pvalb	1.58	0.036
cation binding	10554819	malic enzyme 3, NADP(+)-dependent, mitochondrial	Me3	1.57	0.010
cation binding	10382321	potassium inwardly-rectifying channel, subfamily J, member 2	Kcnj2	1.54	0.019
cation binding	10416800	LIM domain only 7	Lmo7	1.51	2.0E−005
cation binding	10427389	pol polyprotein	LOC280487	1.5	0.014

1 Abbreviation: FC, fold change.

**Table 5:** Genes included in KEGG pathways identified in different gene groups related to condition (*acute stress* or *control*) and 5-HTT genotype (*WT* or *KO*) acquired by DAVID tool.

Group - KEGG pathway (relevance parameter)	Transcript	Gene Title	Symbol	FC^1^	Effect	P-Value
*WT stress* vs. *con* - Axon guidance (Gene Count in %: 7.9; EASE score: 0.036)						
	10440344	roundabout homolog 2 (Drosophila)	Robo2	1.79	up	0.014
	10519747	sema domain, immunoglobulin domain (Ig), shortbasic domain, secreted	Sema3e	1.77	up	0.025
	10375175	slit homolog 3 (Drosophila)	Slit3	1.57	up	0.012
*WT stress* vs. *con* - Calcium signaling pathway (Gene Count in %: 7.9; EASE score: 0.066)						
	10461143	cholinergic receptor, muscarinic 1, CNS	Chrm1	1.87	up	0.011
	10437629	glutamate receptor, ionotropic, NMDA2A (epsilon 1)	Grin2a	1.8	up	0.038
	10561561	ryanodine receptor 1, skeletal muscle	Ryr1	1.58	up	0.019
*stress KO* vs. *WT* - Neuroactive ligand-receptor interaction (Gene Count in %: 4.4; EASE score: 0.024)						
	10464030	adrenergic receptor, alpha 2a	Adra2a	1.91	up	0.002
	10366707	arginine vasopressin receptor 1A	Avpr1a	1.63	up	0.028
	10437629	glutamate receptor, ionotropic, NMDA2A (epsilon 1)	Grin2a	1.85	down	0.038
	10461143	cholinergic receptor, muscarinic 1, CNS	Chrm1	1.71	down	0.005
*stress KO* vs. *WT* - Calcium signaling pathway (Gene Count in %: 3.3; EASE score: 0.075)						
	10366707	arginine vasopressin receptor 1A	Avpr1a	1.63	up	0.028
	10437629	glutamate receptor, ionotropic, NMDA2A (epsilon 1)	Grin2a	1.85	down	0.038
	10461143	cholinergic receptor, muscarinic 1, CNS	Chrm1	1.71	down	0.005
*con KO* vs. *WT* - Ribosome (Gene Count in %: 13.3; EASE score: 0.016)						
	10562320	predicted gene, ribosomal protein L21 pseudogene	Gm12618	1.5	up	0.012
	10466923	ribosomal protein L26///L26 pseudogene///ENSMUSG00000063754	Rpl26	1.53	up	3.4E−04
	10567211	predicted gene, EG668485	EG668485	1.52	up	1.0E−04

1 Abbreviation: FC, fold change.

Highest enrichment scores combined with highest gene coverage were detected in the four clusters glycoprotein, plasma membrane, membrane, and transport in *KO* mice after acute stress (*KO stress* vs. *con*), comprising on average 35% of all genes in this group (10–62% in single enrichment terms; Table 1). At the same time a great overlap existed between these four clusters with about two third of genes included in at least two different clusters (23 of 36 genes) and nearly half of the genes in at least three different clusters (16 of 36 genes; Table 3). Examples of genes in this category were transthyretin (Ttr), klotho (Kl), annexin A1 (Anxa1), angiotensin I converting enzyme 1 (Ace), as well as different channels and receptors like chloride intracellular channel 6 (Clic6), leptin receptor (Lepr) or prolactin receptor (Prlr).

In the direct 5-HTT genotype comparison after acute stress, *stress KO* vs. *WT*, a lower gene coverage of clusters was detected (3–12% in single enrichment terms; Table 1). The first cluster calcium ion binding represented genes like Anxa1, Cdh9, Cabp1, Pvalb, and S100 calcium binding protein A11 (S100a11). The greatly overlapping second and third clusters cilium and cell projection part represented genes, like dynein, axonemal, heavy chain 3 (Dnahc3), radial spoke head 4 homolog A (Rsph4a), Anxa1, Grin2a and Pvalb (Table 4). Of these, genes of the cluster cilium like Dnahc3, Rsph4a, or Anxa1 were upregulated in *KO* mice while genes of the cluster cation binding/calcium ion binding like Grin2a, Cdh9, Cabp1, or Pvalb were upregulated in *WT* mice (Table 4). Pathway analysis slightly supported these results disclosing two highly overlapping KEGG pathways, neuroactive ligand-receptor interaction and calcium signaling pathway, that comprised the genes adrenergic receptor alpha 2a (Adra2a), arginine vasopressin receptor 1A (Avpr1a), cholinergic receptor, muscarinic 1, CNS (Chrm1) and Grin2a (Table 5). The first two, Adra2a and Avpr1a, were found upregulated in *KO* mice, the latter two, Chrm1 and Grin2a in *WT* mice after the acute stress.

In contrast to all these findings related to direct 5-HTT genotype comparison after acute stress, the control condition revealed only minor differences between the *WT* and *KO* mice (*con KO* vs. *WT*). While no functional cluster could be identified in the small group of only 15 differentially regulated genes (Table 1), KEGG pathway analysis discovered the term ribosome, which included three predicted genes or pseudogenes, respectively (GM12618, EG668485, and ribosomal protein L26 pseudogene Rpl26; Table 5).

### Validation of Microarray Results

For validation of the microarray results via quantitative real-time PCR genes Robo2, Cdh9, Ace, Anxa1, and Adra2a were chosen: Robo2 and Cdh9 as representatives of upregulation of functional groups, neurogenesis, axon guidance, and cation binding in *WT* mice after acute stress; Ace and Anxa1 as representatives of upregulation of functional groups, glycoprotein and membrane in *KO* mice after acute stress (Tables 2, 3, 5). Cdh9 and Anxa1 were also representatives of the cluster calcium ion/cation binding and cilium in the *KO* vs. *WT* group after stress as well as Adra2a of the pathway neuroactive ligand-receptor interaction (Tables 4, 5). Real-time PCR data confirmed the microarray data for all investigated genes as the direction of regulation was always identical between Affymetrix array and Taqman assay and resulted in similar fold change values in the above described comparisons. However, in two comparisons the genes Robo2 and Cdh9 did not reach significance in Taqman analysis in the group *WT stress* vs. *con* (for detailed results of all comparisons see supplemental Table S2).

## Discussion

Our study revealed that acute stress triggered immediate changes in the amygdala mRNA profiles compared to the control situation in mice. This is in line with an increasing number of studies reporting expression of genes as determinants for altered neuronal activity in the amygdala or other stress-related brain circuits following various stressors (e.g. [23], [24], [33], [34], [35]). A main finding of our study was, however, that even the very short period of only minutes was sufficient for a modified gene regulation leading to measurable changes in the genome-wide expression profiles. This time span resembles that expected for immediate-early or primary response genes (PRGs) that are induced within 5–10 min of stimulation and do not require *de novo* protein synthesis ([36], review by [37]). PRGs are expressed in the first wave of processes in response to stimulation and involve regulation of for example cellular differentiation, plasticity, transcription, signaling, metabolism, or transport [37], [38]. Concordant to this, our present gene lists also revealed axon guidance, calcium signaling, neuroactive ligand receptor interaction, neurogenesis, cation binding, glycoprotein, membrane, cilium, cell projection part, and transport as enriched pathways or functional clusters related to acute stress response. PRGs are described to be immediately followed by regulatory mechanisms involving transcriptional, mediator-dependent or signal-induced regulation as well as changes in chromatin architecture or microRNA down-regulation to control the commonly transient but sometimes also sustained PRG signaling [37]. By limiting the time span in our experiments from the onset of stress to completed amygdala dissection and RNA stabilization to only 9 min, assumedly most of the detected differentially expressed genes might belong to the group of PRGs and not to the subsequently expressed group of genes that controls them. This is unlike other studies that prepared tissue for RNA extraction at least half an hour after the onset of stress (e.g. [15], [24], [34], [35]) and therefore might disclose a mixture of transient and sustained PRGs together with “control genes”. Our study hereby revealed a different and new set of PRGs involved in the mediation of acute stress in mice. However, further studies are needed to elucidate potentially regulatory mechanisms following the first wave of response to the acute stressor of 1 min exposure to water used here.

Another main finding of our study was that distinct differences existed between mice of the used 5-HTT model (on C57BL/6J background) in a genotype-dependent manner. Importantly, we could confirm previously described differences in anxiety-related behavior and activity between our *WT* and *KO* mice by means of testing in the EPM. This underlines the functional consequences of the gene deletion in challenging situations in the experimental animals. In contrast, we demonstrated that basal corticosterone titers measured under non-stressed conditions did not differ between genotypes, indicating comparable functioning of the hypothalamo-pituitary-adrenocortical stress axis. Corticosterone stress hormone concentrations were expected to increase with a delay of at least 3 min [39]. Albeit not significantly different from basal values, we found a tendency for increased corticosterone titers already within 160 sec related to the acute stress. Corticosteroid-binding globulin may regulate availability of free floating plasma corticosterone and thus provide a mechanism to provide corticosterone immediate after the onset of a stressor [40]. The highest corticosterone titers were found in stressed *KO* mice which are, in accordance with increased anxiety, possibly more primed to expect stressful situations.

In addition, different expression profiles were detected between *WT* and *KO* mice after acute stress without any overlap in the respective gene lists indicating different ways of stress response dependent on 5-HTT protein level. This is consistent with an increasing number of studies revealing altered stress response and coping abilities in 5-HTT *KO* mice compared to their wild-type counterparts (e.g., [13], [14], [15], [16]) and also with our finding of different anxiety-related behavior in the challenging situation of the EPM (mentioned above). Further, in the present study different pathways and functional clusters were detected in *WT* and *KO* mice that are discussed in detail below.

### Response to Acute Stressor in 5-HTT *WT* Mice

Axon guidance/neurogenesis was found enriched only in *WTs* after acute stress, including members of the Slit/Robo family (Slit3, Robo2) and the semaphorins and receptors family (Sema3e). These candidates are assumed to play a role not only in axonal pathfinding and neuronal migration during brain development [41], [42] but also in neuroanatomical connectivity of functional interacting brain regions in the adult brain [43]. In addition, genes of the functional pathways or clusters calcium signaling and calcium/cation binding like Grin2a, Nr4a2, Nr4a3, Chrm1, Ryr1, Cabp1, or Pvalb were upregulated in acutely stressed *WTs*. Several of these genes were shown to be related to spatial memory or memory formation (e.g., Grin2a [44], Nr4a2 [45], Ryr1 [46]). Further the calcium signaling pathway on the whole has been implicated in adaptive responses to synaptic activity, synaptic plasticity, and long-lasting changes in synaptic efficacy and memory (recent review by [47]). Rapid upregulation of all these genes – comprising about 50% of the sum of genes differentially regulated in our stressed *WTs* (20 of 38 genes) – might therefore reflect processes that mediate adaptation to stress involving neuroplasticity to prepare the individuals for future stressful events also in our *WT* mice. This is in line with findings in C57BL/6J mice showing an effective mobilization of plasticity genes in the amygdala which corresponded to an active behavioral response to repeated restraint stress [27].

Beside this group of genes several other appear noticeable, in particular Ntng2 and Adra2a, that were both involved in stress response, but in a somehow unexpected way. Ntng2 knockout mice performed normally in a behavioral test battery and indicated no sign of any overarching defects in the inner ear or auditory nerve but did not startle to an acoustic stimulus [48]. Increased expression of Ntng2 as observed in our *WTs* after acute stress in turn therefore might reflect the preparation of the organism to an increased startle response after an acute stressor which resembles a sensitization or increased arousal or fear. A comparable conclusion can be drawn for Adra2a that modulates synaptic transmission via inactivation of adenyl cyclase and decrease of cAMP in response to catecholamines in neuronal circuits that are correlated with stress *in vivo* [49]. A rapid downregulation as observed in our stressed *WTs* probably reduces these inhibitory effects, resulting in an enhanced neurotransmitter release and therefore a more vigorous arousal or fear and stress reaction. Both candidates therefore seem to strengthen or prolong the response to the acute stressor which might reflect an adaptive way adopted by the *WTs* that might be related with or even necessary for upregulation and mobilization of genes needed for plasticity. Support for this hypothesis comes from studies demonstrating gene expression opposite to that observed in our *WT* mice only in conjunction with pathophysiology: decreased Ntng2 mRNA expression in schizophrenia and bipolar disorder [50] and increased Adra2a expression after chronic psychosocial stress as trigger for depression [51]. However, future studies are required to shed more light on this speculative aspect.

### Response to Acute Stressor in 5-HTT *KO* Mice

In *KO* mice functional pathways/clusters neuroactive ligand receptor interaction, glycoprotein, membrane, transport, cilium, and cell projection were found enriched after acute stress, including genes like Grin2a, Chrm1, Cabp1, Pvalb, Ttr, Kl, Clic6, Prlr, Ace, Adra2a, and Anxa1. For the first four genes, all involved in calcium signaling, a lack of immediate upregulation was observed compared to stressed *WT* mice, leading to the assumption of reduced calcium signaling processes. Thereby *KO* mice might have a reduced ability to adapt to a stressor via neuroplasticity processes as it was supposed above for *WTs* [44], [47]. Alteration or dysfunction of processes involving plasticity and learning to cope with similar situations in the future might be related to maladaptive reactions and the transition to pathological emotion processing as reviewed recently [52], [53], [54]. This might be reflected by our data in two ways. First, several of the genes we identified as rapidly upregulated in acutely stressed *KO*s are concordant to genes upregulated in chronically stressed mice, e.g. Adra2a as mentioned above [51]. Ttr, the top hit in our *KO* gene list with nearly six-fold upregulation is also the top hit in another study with six-fold upregulation after chronic stress in high analgesia (HA) vs. low analgesia mice [55]. Also Kl, Clic6, Prlr, and sclerostin domain containing 1 could be identified as similarly upregulated between the chronically stressed HA mice [55] and our acutely stressed *KO* mice. 5-HTT *KO* mice therefore appear to react already to an acute stressor in a comparable manner as HA mice react to chronic stress whereas no such overlap could be identified with our *WT* mice.

Ttr, a thyroid hormone-binding protein and carrier of thyroxin and retinol, was also demonstrated as top hit of amygdala genes upregulated after stress and fear in fear conditioning experiments in mice [56] and after chronic NMDA receptor antagonist treatment as model for schizophrenia in rats [57]. Thus, second, several of the genes we detected in stressed *KO* mice might indicate a possible connection with stress-related mental disorders in humans, such as anxiety disorders, depression, or schizophrenia. Indeed, polymorphisms in the Ace gene, which encodes a protein effecting blood pressure, cardiovascular and respiratory function, were shown associated with syndromal panic attacks [58] and elevated serum Ace activity and major depression [59]. Adra2a was found associated with HPA axis overdrive [60], symptoms of autonomic, sympathetic dysfunction [61], and its overexpression to be related with depression and suicidal events (review by [62]) and to be downregulated by antidepressant drug [63]. Kl was identified as rare risk candidate for schizophrenia [64] together with genes such as insulin-like growth factor binding protein 2 or ectonucleotide pyrophosphatase/phosphodiesterase 2, both upregulated in a rat model for schizophrenia [57]. Anxa1, a central player in anti-inflammatory and neuroprotective role of microglia was identified to be more strongly expressed in Alzheimer’s disease [65].

Taken together, upregulation of these genes seems to modify the pathophysiology in humans as well as to impair the way of stress response in our *KO* mice, in particular in combination with the above supposed reduced ability for neuroplasticity processes. This is in line with studies reporting indeed impaired coping abilities and stress adaptation in 5-HTT *KO* mice [13], [14], [15], [16], [66]. Fundamental for such altered processes seems the imbalanced serotonergic system with lowered 5-HTT protein levels in our *KO* mice. Whether this includes acute 5-HTT dysfunction at the time of the stressor or instead developmental differences due to targeted inactivation of 5-HTT function in *KO* mice or an intermixture of both remains unclear. However, developmental differences resulting in different morphology and physiology of the amygdala, e.g. altered dendritic morphology and spine density [12], [67] are most likely to also contribute to the distinct gene expression profile detected in our study. As described previously (review by [12]) also our EPM data indicated *KO* mice as showing more basal anxiety-like behavior compared to *WT* mice. Our study therefore revealed a new set of differentially regulated genes dependent on stressor and particularly 5-HTT genotype. Several of them reflect already known marker/candidate genes for stress-related psychiatric disorders whereas others might serve as new potential candidate genes for e.g. anxiety disorders, depression, or schizophrenia. Future studies however are needed to test for such presumable relations.

### Limitations

In the present study several limitations have to be considered. First of all, no other brain regions in addition to the amygdala were investigated that might have allowed to control the identified effects as relatively specific to the amygdala. In fact, several of the differentially expressed amygdala genes discussed above actually resembled findings from other corticolimbic emotion processing regions such as the prefrontal cortex (e.g., [55]). It is likely, therefore, that other regions are also involved in the appraisal of the stressor and in driving amygdala activity and gene expression, in particular the prefrontal cortex that is functionally tightly connected with the amygdala (e.g., [54]). However, focus on the amygdala might have allowed identifying first PRGs even after few minutes of acute stress response as it is described to produce the most expression changes compared to prefrontal cortex and hippocampus in C57BL/6J mice [27]. Further, no differentiation was performed between amygdala subregions and nuclei such as the basal and lateral amygdala and central amygdaloid or medial amygdaloid nucleus, although they are known to vary in their extrinsic and intra-amygdaloid connections and stress response (review by [33]). We decided to merge all parts as well as amygdalae from left and right hemisphere per mouse to gain enough material from each individual for one microarray-based transcriptome analysis, that is without the need to pool different animals. However, thereby we had to accept an intermixture and weakening of effects. In addition, although mice of both genotypes experienced similar life time events (e.g., weaning, group-housing prior to single housing, EPM), these events might have been perceived differentially depending upon the genotype. Thereby the observed differences may be primed by heightened state anxiety in the *KO* mice. Nevertheless, we identified overall low numbers of differentially regulated genes in the respective group comparisons that were lowest in the control condition (*KO* vs. *WT*: 15 genes) and highest in the genotype comparison after acute stress (90 genes). This might, admittedly, reflect a less optimal gene list as supposed by Huang et al. ([31], 100–2000 genes supposed as optimal), albeit it is in the range of other studies, e.g. Matsuoka et al. ([57], 39 genes). It might, in contrast, also reflect the high standardization of mice before and during the experiments and tissue preparation, that might have reduced or filtered out some of the “background-noise”. Supportive for this might be that several other aspects of our gene list indicate good quality as requested by Huang et al. [31]: many important or marker genes were detected, notable portions of differentially regulated genes are involved in certain interesting biological processes, a high proportion of enriched biology exists using DAVID analysis, and independent quantitative real-time PCR experiments confirmed the investigated marker genes. It should be noted further, that nearly no overlap existed in transcript profiles between our *WT* and *KO* mice, particularly after acute stress. Complete different ways of stress response therefore seem to be adopted dependent on 5-HTT genotype indicating adaptation processes via activated neuroplasticity in *WTs* and impaired plasticity but pathophysiology-related processes in *KO* mice. This is in line with C57BL/6J compared to DBA/2J mice in which stress affected almost entirely different sets of genes including plasticity-related ones in C57BL/6J [27]. Another possibility might be that *KO* and *WT* mice experienced the same acute stressor in different ways, for example with distinctly higher intensity in *KO* mice: The slightly increased corticosterone response in *KO* compared to *WT* mice after stress might serve as subtle hint for this. However, future studies identifying also the behavior during acute stress exposure as well as faster indicators of HPA axis activity (e.g., ACTH response) are needed to elucidate this aspect.

### Conclusions

Overall, time-limited procedure and microarray-based amygdala transcriptome analysis revealed a new set of genes differentially expressed in the first wave response of the amygdala to an acute stress condition in mice. Further, large differences in the mRNA profiles were detected between 5-HTT genotypes involving neuroplasticity and adaptation in *WT* mice but impaired plasticity and processes related to chronic stress and pathophysiology in *KO* mice. This indicates different coping styles dependent upon the availability of 5-HTT protein and accentuates the role of the serotonergic system in the different processing of stressors and threat in the amygdala as a core system of emotion processing. Moreover, several of the genes that we found to be differentially expressed immediate-early after the acute stress-inducing situation in mice might provide promising targets for future therapeutic interventions of stress-related disorders in humans like anxiety disorders or mood disorders.

## Supporting Information

Table S1 Gene list report of differentially regulated amygdala genes in mouse groups related to condition of acute stress or control (*stress* or *con*) and 5-HTT genotype (*WT* or *KO*).
(DOC)

Table S2 Comparison between Array- and Taqman-based gene expression in the genes Robo2, Cdh9, Ace, Anxa1, and Adra2a, showing the direction of regulation (up- or down-regulated), fold changes (FC), and P-values in the group comparisons related to condition (*acute stress* or *control*) and 5-HTT genotype (*WT* or *KO*) of mice.
(DOC)
