# Supplementary material for: Effect of Acute Stressor and Serotonin Transporter Genotype on Amygdala First Wave Transcriptome in Mice
Source: PLoS One. 2013 Mar 11;8(3):e58880. doi: 10.1371/journal.pone.0058880 (PMC3594195; doi:10.1371/journal.pone.0058880)
Supplement: Table S1 — Gene list report of differentially regulated amygdala genes in mouse groups related to condition of acute stress or control (stress or con) and 5-HTT genotype (WT or KO). (DOC) [file pone.0058880.s001.doc]

**Supplemental Table S1.** Gene list report of differentially regulated amygdala genes in mouse groups related to condition of acute stress or control (*stress* or *con*) and 5-HTT genotype (*WT* or *KO*).

| Group | Transcript | Gene Title | Gene Symbol | Fold Change | Regulation | P-Value |
| --- | --- | --- | --- | --- | --- | --- |
| *WT stress vs. con* | 10586591 | carbonic anyhydrase 12 | Car12 | 2.88 | up | 0.032 |
|  | 10523483 | [PR domain containing 8](http://david.abcc.ncifcrf.gov/geneReportFull.jsp?rowids=458549) | prdm8 | 2.25 | up | 0.047 |
|  | 10485745 | anoctamin 3 | Ano3 | 2.09 | up | 0.006 |
|  | 10423230 | cadherin 9 | Cdh9 | 2.07 | up | 0.004 |
|  | 10374012 | RAS-like, family 10, member A | Rasl10a | 2.06 | up | 0.044 |
|  | 10504838 | nuclear receptor subfamily 4, group A, member 3 | Nr4a3 | 2.06 | up | 0.020 |
|  | 10353309 | junctophilin 1 | Jph1 | 2.04 | up | 0.032 |
|  | 10461143 | cholinergic receptor, muscarinic 1, CNS | Chrm1 | 1.87 | up | 0.011 |
|  | 10595094 | RIKEN cDNA 2310046A06 gene | 2310046A06Rik | 1.83 | up | 0.002 |
|  | 10556381 | microtubule associated monoxygenase, calponin and LIM domain containing 2 | Mical2 | 1.81 | up | 0.033 |
|  | 10437629 | glutamate receptor, ionotropic, NMDA2A (epsilon 1) | Grin2a | 1.8 | up | 0.038 |
|  | 10440344 | roundabout homolog 2 (Drosophila) | Robo2 | 1.79 | up | 0.014 |
|  | 10519747 | sema domain, immunoglobulin domain (Ig), short basic domain, secreted, (semaphorin) 3E | Sema3e | 1.77 | up | 0.025 |
|  | 10407126 | polo-like kinase 2 (Drosophila) | Plk2 | 1.75 | up | 0.009 |
|  | 10482772 | nuclear receptor subfamily 4, group A, member 2 | Nr4a2 | 1.73 | up | 0.039 |
|  | 10572130 | lipoprotein lipase; similar to Lipoprotein lipase precursor (LPL) | Lpl | 1.72 | up | 0.027 |
|  | 10481349 | netrin G2 /// RIKEN cDNA 6530402F18 gene | Ntng2 | 1.7 | up | 0.003 |
|  | 10548940 | LIM domain only 3 | Lmo3 | 1.7 | up | 0.002 |
|  | 10495094 | RIKEN cDNA 6530418L21 gene | Fam212b | 1.69 | up | 0.015 |
|  | 10371296 | glycosyltransferase 8 domain containing 2 | Glt8d2 | 1.63 | up | 0.020 |
|  | 10607562 | connector enhancer of kinase suppressor of Ras 2 | Cnksr2 | 1.62 | up | 0.002 |
|  | 10568731 | retinoblastoma 1 | RB1 | 1.60 | up | 0.036 |
|  | 10532956 | calcium binding protein 1 | Cabp1 | 1.59 | up | 0.023 |
|  | 10561561 | ryanodine receptor 1, skeletal muscle | Ryr1 | 1.58 | up | 0.019 |
|  | 10375175 | slit homolog 3 (Drosophila) | Slit3 | 1.57 | up | 0.012 |
|  | 10510643 | pleckstrin homology domain containing, family G (with RhoGef domain) member 5 | Plekhg5 | 1.57 | up | 0.044 |
|  | 10526038 | matrix metallopeptidase 17 | Mmp17 | 1.55 | up | 0.032 |
|  | 10544660 | oxysterol binding protein-like 3 | Osbpl3 | 1.54 | up | 0.032 |
|  | 10435791 | ENSMUSG00000076372; microRNA 568 | Mir568 | 1.54 | up | 0.032 |
|  | 10514240 | solute carrier family 24 (sodium/potassium/calcium exchanger), member 2 | Slc24a2 | 1.54 | up | 0.019 |
|  | 10554819 | malic enzyme 3, NADP(+)-dependent, mitochondrial | Me3 | 1.51 | up | 0.029 |
|  | 10364792 | polo-like kinase 5 (Drosophila) | Plk5 | 1.51 | up | 0.023 |
|  | 10416800 | LIM domain only 7 | Lmo7 | 1.51 | up | 0.005 |
|  | 10533751 | phosphatidylinositol transfer protein, membrane-associated 2 | Pitpnm2 | 1.51 | up | 0.027 |
|  | 10383289 | brain-specific angiogenesis inhibitor 1-associated protein 2 | Baiap2 | 1.51 | up | 0.041 |
|  | 10434845 | interleukin 1 receptor accessory protein | Il1rap | 1.5 | up | 0.049 |
|  | 10464030 | adrenergic receptor, alpha 2a | Adra2a | 1.62 | down | 0.045 |
|  | 10603182 | Rho GTPase activating protein 6 | Arhgap6 | 1.56 | down | 0.048 |

Supplemental Table S1: continued

| *KO stress vs. con* | 10454192 | transthyretin | Ttr | 5.92 | up | 0.024 |
| --- | --- | --- | --- | --- | --- | --- |
|  | 10356403 | potassium inwardly-rectifying channel, subfamily J, member 13 | Kcnj13 | 5.82 | up | 0.007 |
|  | 10527870 | klotho | Kl | 3.45 | up | 0.015 |
|  | 10351224 | coagulation factor V; similar to Murine coagulation factor V | F5 | 2.83 | up | 0.016 |
|  | 10395389 | sclerostin domain containing 1 | Sostdc1 | 2.71 | up | 0.028 |
|  | 10543921 | solute carrier family 13 (sodium/sulfate symporters), member 4 | Slc13a4 | 2.38 | up | 0.025 |
|  | 10478048 | lipopolysaccharide binding protein | Lbp | 2.38 | up | 0.040 |
|  | 10539393 | solute carrier family 4, sodium bicarbonate cotransporter, member 5 | Slc4a5 | 2.28 | up | 0.027 |
|  | 10547191 | transmembrane protein 72 | Tmem72 | 2.1 | up | 0.033 |
|  | 10347277 | insulin-like growth factor binding protein 2 | Igfbp2 | 2.06 | up | 0.032 |
|  | 10569344 | insulin-like growth factor 2 | Igf2 | 2.05 | up | 0.043 |
|  | 10436947 | potassium voltage-gated channel, Isk-related subfamily, gene 2 | Kcne2 | 2.04 | up | 0.035 |
|  | 10495712 | ATP-binding cassette, sub-family A (ABC1), member 4 | Abca4 | 2.04 | up | 0.029 |
|  | 10440091 | collagen, type VIII, alpha 1 | Col8a1 | 2.01 | up | 0.021 |
|  | 10436958 | chloride intracellular channel 6 | Clic6 | 1.98 | up | 0.003 |
|  | 10600024 | G-protein-coupled receptor 50 | Gpr50 | 1.95 | up | 0.050 |
|  | 10602033 | claudin 2 | Cldn2 | 1.94 | up | 0.023 |
|  | 10428619 | ectonucleotide pyrophosphatase/phosphodiesterase 2 | Enpp2 | 1.92 | up | 0.031 |
|  | 10362104 | solute carrier family 2 (facilitated glucose transporter), member 12 | Slc2a12 | 1.91 | up | 0.016 |
|  | 10530269 | RNA binding motif protein 47 | Rbm47 | 1.9 | up | 0.034 |
|  | 10456005 | CD74 antigen (invariant polypeptide of major histocompatibility complex, class II antigen-associated) | Cd74 | 1.87 | up | 0.021 |
|  | 10506301 | leptin receptor | Lepr | 1.86 | up | 0.034 |
|  | 10450154 | histocompatibility 2, class II antigen A, alpha | H2-Aa | 1.85 | up | 0.019 |
|  | 10466606 | annexin A1 | Anxa1 | 1.85 | up | 0.023 |
|  | 10381962 | angiotensin I converting enzyme (peptidyl-dipeptidase A) 1 | Ace | 1.81 | up | 0.016 |
|  | 10423049 | prolactin receptor | Prlr | 1.76 | up | 0.035 |
|  | 10357870 | proline arginine-rich end leucine-rich repeat | Prelp | 1.69 | up | 0.048 |
|  | 10362896 | CD24a antigen | Cd24a | 1.68 | up | 0.044 |
|  | 10542993 | paraoxonase 3 | Pon3 | 1.66 | up | 0.036 |
|  | 10532839 | transient receptor potential cation channel, subfamily V, member 4 | Trpv4 | 1.65 | up | 0.039 |
|  | 10538459 | aquaporin 1 | Aqp1 | 1.61 | up | 0.040 |
|  | 10422728 | disabled homolog 2 (Drosophila) | Dab2 | 1.59 | up | 0.026 |
|  | 10373588 | retinol dehydrogenase 5 | Rdh5 | 1.58 | up | 0.015 |
|  | 10344897 | sulfatase 1 | Sulf1 | 1.57 | up | 0.034 |
|  | 10529651 | homeobox, msh-like 1 | Msx1 | 1.54 | up | 0.033 |
|  | 10351504 | expressed sequence AI506816 | AI506816 | 1.52 | up | 0.034 |
|  | 10541354 | alpha-2-macroglobulin | A2m | 1.52 | up | 0.048 |
|  | 10511180 | matrix-remodelling associated 8 | Mxra8 | 1.52 | up | 0.006 |
|  | 10584653 | C1q and tumor necrosis factor related protein 5 /// membrane-type frizzled-related protein | C1qtnf5 /// Mfrp | 1.51 | up | 0.028 |
|  | 10600093 | zinc finger protein 185 | Zfp185 | 1.51 | up | 0.039 |
|  | 10536220 | collagen, type I, alpha 2 | Col1a2 | 1.5 | up | 0.034 |

Supplemental Table S1: continued

|  | 10366391 | potassium voltage gated channel, Shaw-related subfamily, member 2 | Kcnc2 | 1.54 | down | 0.030 |
| --- | --- | --- | --- | --- | --- | --- |
| *con KO vs. WT* | 10378816 | solute carrier family 6 (neurotransmitter transporter, serotonin), member 4 | Slc6a4 | 2.91 | up | 0.002 |
|  | 10367024 | tachykinin 2 | Tac2 | 2.12 | up | 0.048 |
|  | 10594963 | unc-13 homolog C (C. elegans) | Unc13c | 2.05 | up | 0.035 |
|  | 10466923 | ribosomal protein L26 /// ribosomal protein L26 pseudogene /// ENSMUSG00000063754 | Rpl26 | 1.53 | up | 3.4E-04 |
|  | 10567211 | predicted gene, EG668485 | EG668485 | 1.52 | up | 1.0E-04 |
|  | 10598023 | ENSMUSG00000064337 | mt-Rnr1 | 1.51 | up | 0.017 |
|  | 10562320 | predicted gene, ribosomal protein L21 pseudogene | Gm12618 | 1.5 | up | 0.012 |
|  | 10356403 | potassium inwardly-rectifying channel, subfamily J, member 13 | Kcnj13 | 2.05 | down | 0.010 |
|  | 10454731 | nuclear encoded rRNA 5S; | n-R5s | 1.87 | down | 0.017 |
|  | 10583203 | per-hexamer repeat gene 4 | Phxr4 | 1.64 | down | 0.031 |
|  | 10398342 | retrotransposon-like 1; RIKEN cDNA 6430411K18 gene | RTL1 | 1.58 | down | 0.028 |
|  | 10389699 | ENSMUSG00000056668 | ENSMUSG00000056668 | 1.57 | down | 0.026 |
|  | 10398396 | ENSMUSG00000076145 | Mir679 | 1.54 | down | 0.033 |
|  | 10510090 | predicted gene 13103 | Gm13103 | 1.52 | down | 0.022 |
|  | 10557853 | RIKEN cDNA B230325K18 gene | B230325K18Rik | 1.51 | down | 0.048 |
|  | 10374350 | U6 small nuclear RNA | Rnu6 | 1.5 | down | 0.039 |
| *stress KO vs. WT* | 10378816 | solute carrier family 6 (neurotransmitter transporter, serotonin), member 4 | Slc6a4 | 3.69 | up | 2.8E-09 |
|  | 10567452 | dynein, axonemal, heavy chain 3 | Dnahc3 | 2.84 | up | 0.009 |
|  | 10358476 | proteoglycan 4 (megakaryocyte stimulating factor, articular superficial zone protein) | Prg4 | 2.63 | up | 0.038 |
|  | 10386211 | RIKEN cDNA 3100002J23 gene | FAM183B | 2.00 | up | 0.004 |
|  | 10362472 | radial spoke head 4 homolog A (Chlamydomonas) | rsph4a | 1.99 | up | 0.010 |
|  | 10504664 | ENSMUSG00000078708 | ENSMUSG00000078708 | 1.96 | up | 0.016 |
|  | 10472809 | distal-less homeobox 1 | Dlx1 | 1.94 | up | 7.4E-04 |
|  | 10464030 | adrenergic receptor, alpha 2a | Adra2a | 1.91 | up | 0.002 |
|  | 10481272 | RIKEN cDNA 1700007K13 gene | 1700007K13Rik | 1.91 | up | 0.029 |
|  | 10599812 | zinc finger protein of the cerebellum 3 | Zic3 | 1.88 | up | 0.018 |
|  | 10604576 | glypican 3 | Gpc3 | 1.87 | up | 0.001 |
|  | 10401708 | neuroglobin | Ngb | 1.86 | up | 0.013 |
|  | 10389759 | ankyrin-repeat and fibronectin type III domain containing 1 | Ankfn1 | 1.86 | up | 0.003 |
|  | 10360463 | phospholipase D family, member 5 | Pld5 | 1.86 | up | 0.028 |
|  | 10466606 | annexin A1 | Anxa1 | 1.85 | up | 0.030 |
|  | 10427661 | ENSMUSG00000063249 | ENSMUSG00000063249 | 1.82 | up | 0.039 |
|  | 10602688 | ubiquitin specific protease 51 | USP51 | 1.79 | up | 0.010 |
|  | 10431558 | outer dense fiber of sperm tails 3B | Odf3b | 1.76 | up | 0.015 |
|  | 10374777 | epidermal growth factor-containing fibulin-like extracellular matrix protein 1 | Efemp1 | 1.74 | up | 0.036 |
|  | 10399943 | RIKEN cDNA 1110049B09 gene; Cadherin-like protein 28 | CDHR3 | 1.74 | up | 0.037 |
|  | 10427681 | sperm flagellar 2 | SPEF2 | 1.72 | up | 0.027 |
|  | 10362896 | CD24a antigen | Cd24a | 1.70 | up | 0.008 |
|  | 10389754 | RIKEN cDNA 4932411E22 gene | 4932411E22Rik | 1.68 | up | 0.003 |
|  | 10457587 | zinc finger protein 521 | Zfp521 | 1.68 | up | 0.011 |

Supplemental Table S1: continued

|  | 10402211 | fibulin 5 | Fbln5 | 1.67 | up | 0.007 |
| --- | --- | --- | --- | --- | --- | --- |
|  | 10494684 | sperm associated antigen 17 | Spag17 | 1.65 | up | 0.003 |
|  | 10483626 | distal-less homeobox 2 | Dlx2 | 1.65 | up | 0.003 |
|  | 10603182 | Rho GTPase activating protein 6 | Arhgap6 | 1.64 | up | 0.006 |
|  | 10566730 | serine/threonine kinase 33 | Stk33 | 1.63 | up | 0.006 |
|  | 10366707 | arginine vasopressin receptor 1A | Avpr1a | 1.63 | up | 0.028 |
|  | 10406845 | forkhead box D1 | Foxd1 | 1.62 | up | 0.048 |
|  | 10356512 | IQ motif containing with AAA domain | Iqca | 1.62 | up | 0.035 |
|  | 10457640 | S100 calcium binding protein A11 (calgizzarin) | S100a11 | 1.61 | up | 0.047 |
|  | 10491993 | stomatin (Epb7.2)-like 3 | Stoml3 | 1.6 | up | 0.031 |
|  | 10464370 | solute carrier family 18 (vesicular monoamine), member 2 | Slc18a2 | 1.6 | up | 0.020 |
|  | 10547227 | ret proto-oncogene | Ret | 1.59 | up | 0.006 |
|  | 10416340 | glial cell line derived neurotrophic factor family receptor alpha 2 | Gfra2 | 1.59 | up | 0.003 |
|  | 10376956 | heparan sulfate (glucosamine) 3-O-sulfotransferase 3°1 | Hs3st3a1 | 1.58 | up | 0.016 |
|  | 10567450 | ENSMUSG00000078596 | ENSMUSG00000078596 | 1.57 | up | 0.012 |
|  | 10400304 | EGL nine homolog 3 (C. elegans) | Egln3 | 1.56 | up | 0.046 |
|  | 10565463 | coiled-coil domain containing 81 | Ccdc81 | 1.56 | up | 0.009 |
|  | 10524310 | tetratricopeptide repeat domain 28 | Ttc28 | 1.55 | up | 0.036 |
|  | 10453166 | cyclin-dependent kinase-like 4 | Cdkl4 | 1.55 | up | 0.028 |
|  | 10375137 | potassium large conductance calcium-activated channel, subfamily M, beta member 1 | Kcnmb1 | 1.54 | up | 0.032 |
|  | 10449741 | salt inducible kinase 1 | Sik1 | 1.54 | up | 0.015 |
|  | 10477986 | neuronatin | Nnat | 1.53 | up | 0.050 |
|  | 10518352 | predicted gene; similar to High mobility group box 2 | Hmgb2 | 1.52 | up | 0.005 |
|  | 10452419 | ephrin A5 | Efna5 | 1.52 | up | 0.026 |
|  | 10586907 | meiosis-specific nuclear structural protein 1 | Mns1 | 1.51 | up | 0.033 |
|  | 10423505 | carboxymethylenebutenolidase-like (Pseudomonas) | Cmbl | 1.51 | up | 0.024 |
|  | 10541885 | sodium channel, nonvoltage-gated 1 alpha | Scnn1a | 1.51 | up | 0.018 |
|  | 10458052 | erythrocyte protein band 4.1-like 4a | Epb4.1l4a | 1.5 | up | 0.026 |
|  | 10358457 | predicted gene 4322; brain expressed gene 4 | Bex4 | 1.5 | up | 0.038 |
|  | 10353010 | myeloblastosis oncogene-like 1 | Mybl1 | 1.5 | up | 0.047 |
|  | 10440329 | RIKEN cDNA 9330155M09 gene | 9330155M09Rik | 2.13 | down | 0.024 |
|  | 10521811 | ENSMUSG00000057577 | Gm10025 | 2.04 | down | 0.016 |
|  | 10506431 | ENSMUSG00000070886 | Gm10304 | 2.01 | down | 0.044 |
|  | 10506452 | cDNA sequence AY512949 | AY512949 | 1.99 | down | 0.039 |
|  | 10504137 | ENSMUSG00000073875 | Gm10595 | 1.95 | down | 0.031 |
|  | 10504201 | ENSMUSG00000073868 | Gm10590 | 1.95 | down | 0.031 |
|  | 10512350 | ENSMUSG00000078243 | AC087559.2 | 1.95 | down | 0.031 |
|  | 10454731 | nuclear encoded rRNA 5S | n-R5s | 1.89 | down | 0.020 |
|  | 10545096 | predicted gene 15072; RIKEN cDNA 2410003J06 gene | Mageb16 | 1.88 | down | 0.018 |
|  | 10437629 | glutamate receptor, ionotropic, NMDA2A (epsilon 1) | Grin2a | 1.85 | down | 0.038 |
|  | 10423230 | cadherin 9 | Cdh9 | 1.84 | down | 0.023 |

Supplemental Table S1: continued

|  | 10386705 | ring finger protein 112 | Zfp179 | 1.75 | down | 0.022 |
| --- | --- | --- | --- | --- | --- | --- |
|  | 10542221 | ENSMUSG00000076212 | Mir680-1 | 1.73 | down | 0.038 |
|  | 10485745 | anoctamin 3 | Ano3 | 1.73 | down | 0.001 |
|  | 10551250 | ENSMUSG00000077509 | AC120367.1 | 1.72 | down | 0.036 |
|  | 10461143 | cholinergic receptor, muscarinic 1, CNS | Chrm1 | 1.71 | down | 0.005 |
|  | 10556381 | microtubule associated monoxygenase, calponin and LIM domain containing 2 | Mical2 | 1.69 | down | 0.047 |
|  | 10520351 | RIKEN cDNA B930011P16 gene | B930011P16Rik | 1.67 | down | 0.037 |
|  | 10495094 | RIKEN cDNA 6530418L21 gene | 6530418L21Rik | 1.66 | down | 0.010 |
|  | 10595626 | Small nucleolar RNA SNORD50 | SNORD50 | 1.66 | down | 0.007 |
|  | 10495621 | ENSMUSG00000065569 | Mir137 | 1.63 | down | 0.020 |
|  | 10423791 | RIKEN cDNA 4930447A16 gene | 4930447A16Rik | 1.6 | down | 0.033 |
|  | 10532956 | calcium binding protein 1 | Cabp1 | 1.58 | down | 0.009 |
|  | 10430297 | parvalbumin | Pvalb | 1.58 | down | 0.036 |
|  | 10554819 | malic enzyme 3, NADP(+)-dependent, mitochondrial | Me3 | 1.57 | down | 0.010 |
|  | 10514240 | solute carrier family 24 (sodium/potassium/calcium exchanger), member 2 | Slc24a2 | 1.56 | down | 0.002 |
|  | 10399965 | RIKEN cDNA F730043M19 gene | F730043M19Rik | 1.56 | down | 0.013 |
|  | 10382321 | potassium inwardly-rectifying channel, subfamily J, member 2 | Kcnj2 | 1.54 | down | 0.019 |
|  | 10510643 | pleckstrin homology domain containing, family G (with RhoGef domain) member 5 | Plekhg5 | 1.54 | down | 0.049 |
|  | 10516227 | RIKEN cDNA 3100002H09 gene | 3100002H09Rik | 1.53 | down | 0.025 |
|  | 10583203 | per-hexamer repeat gene 4 | Phxr4 | 1.52 | down | 0.049 |
|  | 10557175 | U6 small nuclear RNA | Rnu6 | 1.51 | down | 0.006 |
|  | 10416800 | LIM domain only 7 | Lmo7 | 1.51 | down | 2.0E-05 |
|  | 10586166 | Small nucleolar RNA SNORD18 | SNORD18 | 1.5 | down | 0.011 |
|  | 10374352 | predicted gene 4638 | Gm4638 | 1.5 | down | 0.014 |
|  | 10427389 | pol polyprotein | LOC280487 | 1.5 | down | 0.014 |
