# Supplementary material for: Effect of Acute Stressor and Serotonin Transporter Genotype on Amygdala First Wave Transcriptome in Mice
Source: PLoS One. 2013 Mar 11;8(3):e58880. doi: 10.1371/journal.pone.0058880 (PMC3594195; doi:10.1371/journal.pone.0058880)
Supplement: Table S2 — Comparison between Array- and Taqman-based gene expression in the genes Robo2, Cdh9, Ace, Anxa1, and Adra2a, showing the direction of regulation (up- or down-regulated), fold changes (FC), and P-values in the group comparisons related to condition (acute stress or control) and 5-HTT genotype (WT or KO) of mice. (DOC) [file pone.0058880.s002.doc]

**Supplemental Table S2.** Comparison between Array- and Taqman-based gene expression in the genes Robo2, Cdh9, Ace, Anxa1, and Adra2a, showing the direction of regulation (up- or down-regulated), fold changes (FC), and P-values in the group comparisons related to condition (*acute stress* or *control*) and 5-HTT genotype (*WT* or *KO*) of mice.

| **Gene** | **Method** | ***WT stress* vs. *con*** | ***KO stress* vs. *con*** | ***stress KO* vs. *WT*** |
| --- | --- | --- | --- | --- |
| Robo2 | Affymetrix Array^1^ | **up** (FC 1.79, P = 0.014) | n. diff. | n. diff. |
|  | Taqman assay^2^ | up (FC 1.87, P =0.18) | n. diff. | n. diff. |
|  |  |  |  |  |
| Cdh9 | Affymetrix Array^1^ | **up** (FC 2.07, P = 0.004) | n. diff. | **down** (FC 1.84, P = 0.023) |
|  | Taqman assay^2^ | up (FC 1.60, P = 0.25) | n. diff. | **down** (FC 2.22, P = 0.028) |
|  |  |  |  |  |
| Ace | Affymetrix Array^1^ | n. diff. | **up** (FC 1.81, P = 0.016) | n. diff. |
|  | Taqman assay^2^ | n. diff. | **up** (FC 2.79, P = 0.016) | n. diff. |
|  |  |  |  |  |
| Anxa1 | Affymetrix Array^1^ | n. diff. | **up** (FC 1.85, P = 0.023) | **up** (FC 1.85, P = 0.030) |
|  | Taqman assay^2^ | **down** (FC 2.57, P = 0.028) | **up** (FC 2.15, P = 0.047) | **up** (FC 3.23, P = 0.028) |
|  |  |  |  |  |
| Adra2a | Affymetrix Array^1^ | **down** (FC 1.62, P = 0.045) | n. diff. | **up** (FC 1.91, P = 0.002) |
|  | Taqman assay^2^ | **down** (FC 2.78, P = 0.047) | n. diff. | **up** (FC 2.06, P = 0.009) |

^1^Parametric unpaired T-test; ^2^nonparametric Mann-Whitney U Test; n. diff.: not differentially regulated (fold change < 1.5 and/or P > 0.05); bold letters indicate statistically significant differences at P < 0.05.
